# A Rare Organism Causing Cholecystitis With Bacteremia in a Breast Cancer Patient

**DOI:** 10.7759/cureus.54549

**Published:** 2024-02-20

**Authors:** Mina Ghaly, Alyssa Zakala, Kavya Penmethsa, Kristy D Johnson-Pich

**Affiliations:** 1 Internal Medicine, Southeast Health Medical Center, Dothan, USA; 2 Research, Alabama College of Osteopathic Medicine, Dothan, USA

**Keywords:** cholecystitis/diagnosis, rare pathogen, levan, anaerobic bacteremia, pantoea infection, pantoea

## Abstract

*Pantoea*, a gram-negative, rod-shaped, anaerobic bacterium, is a rare cause of human disease. *Pantoea* species have been known to mostly cause pulmonary disease in agricultural workers as they are native to select crops and wild animal furs. However, in very few documented cases, *Pantoea* has been discovered as the source of nosocomial infections, usually in the setting of an immunocompromised host. This case report details the clinical course of a 62-year-old immunocompromised female with stage 3 breast cancer presenting with acute cholecystitis and bacteremia and the unexpected discovery of *Pantoea* in peripheral and chemotherapy port blood cultures. After appropriate management and susceptibility testing, the patient fortunately recovered with initial cefepime and eventual levofloxacin to target the *Pantoea* species. To our knowledge, this is the third documented case worldwide of *Pantoea* isolated from cholecystitis with associated bacteremia and the first documented case in North America. Of special interest, a few months after her infection, the patient was found to be free of breast cancer. *Pantoea* species are known to contain levan, an exopolysaccharide, that has been seen to upregulate tumor suppressor genes. This should be considered in the future management and research of *Pantoea* infections.

## Introduction

Biliary tract infections, such as cholecystitis, are a common cause of bacteremia. Most bacteria isolated from these infections are gram-negative organisms like *Escherichia coli* and *Klebsiella pneumoniae* [1]. In this case report, we introduce a 62-year-old immunocompromised female with stage 3 breast cancer who presents with acute cholecystitis and associated bacteremia. During her hospital stay, blood cultures were obtained, and a rare anaerobic pathogen, *Pantoea*, was identified as the causative organism. 

*Pantoea* is a genus of yellow-pigmented, gram-negative, rod-shaped, anaerobic bacteria that is native to environments such as plant surfaces, animal coats, and human feces [2]. As a gram-negative bacterium, its lipopolysaccharide (LPS) endotoxin is what drives illness in humans [3]. Regarding human disease, *Pantoea* is typically associated with occupational-related disorders, especially in the agricultural industry. For example, *Pantoea* and its endotoxins have been identified in cotton plants, and inhalation of cotton dust by workers can manifest as a respiratory disorder known as byssinosis [3]. 

In healthcare or hospital-acquired infections, *Pantoea* is rarely identified as the causative organism. However, it has the potential to cause opportunistic infection, especially in immunocompromised patients [4]. In nosocomial infections, several species of *Pantoea* have been implicated as a rare cause of bacteremia, cholangitis, and peritonitis. Relevant literature states that while cases of *Pantoea* bacteremia are rare, they can be traced back to infection of medical devices, such as indwelling catheters and central lines [2]. For our patient in this report, blood cultures were obtained from her chemotherapy port as well as peripherally grown *Pantoea*. Her chemotherapy port was believed to be the source of infection.

## Case presentation

A 62-year-old immunocompromised female with a medical history significant for stage 3 breast cancer, insulin-dependent type 2 diabetes mellitus, and a previous brain tumor treated with excision several years prior was undergoing her weekly outpatient chemotherapy in the early morning when she suddenly experienced dizziness, shortness of breath, and generalized weakness. The technician discontinued chemotherapy shortly after finding the patient to be hypotensive and tachycardic, thus prompting the transportation of the patient to the emergency room for further evaluation and management. 

**Figure 1:**
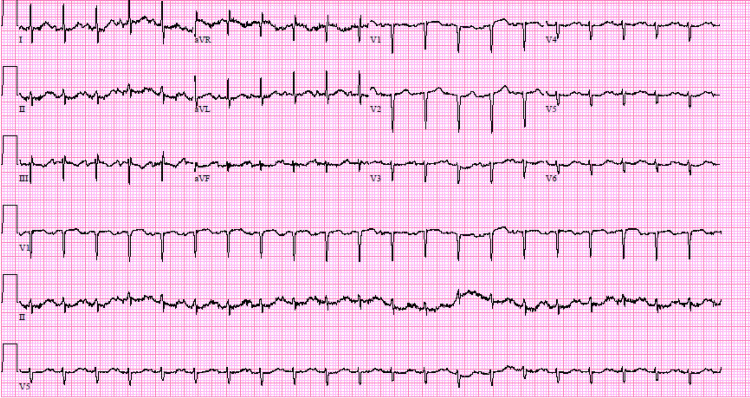
ECG immediately obtained in the emergency department displays sinus tachycardia with left-axis deviation and no definitive ischemia. Wandering baseline present in several leads is an artifact of motion. Serum troponins were initially elevated at 85 but trended down to 58 on repeat lab. A pulmonary embolism was considered due to the patient's sinus tachycardia, dyspnea, hypoxia, and hypercoagulable state given her breast cancer status; however, a subsequent CT of the chest with IV contrast was essential to ruling this out. ECG: electrocardiogram; CT: computed tomography

**Figure 2:**
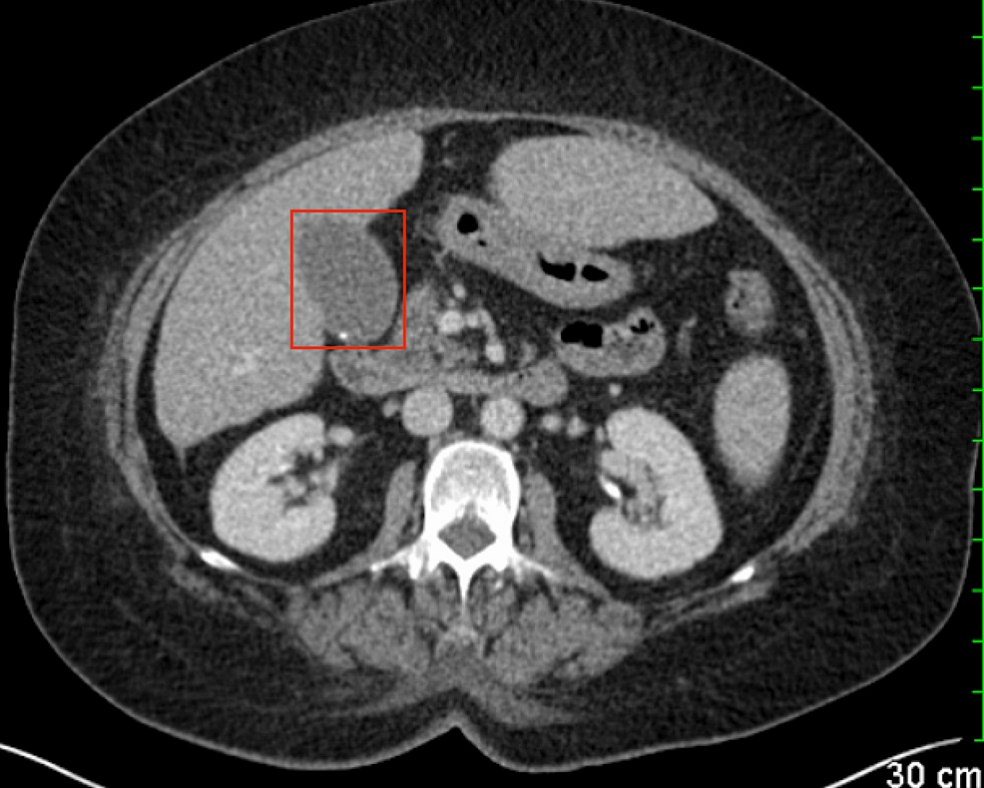
CT slice at the level of the abdomen reveals cholelithiasis. The gallbladder is outlined in the red box showing a hyperdense gallstone in the dependent portion of the gallbladder. CT: computed tomography

Upon arrival at the emergency department, the patient was found to be hypotensive with a blood pressure at 77/51 mmHg along with an oxygen saturation in the upper 80s on room air. She was given a total of 3 L of IV fluids and placed on a 2 L nasal cannula, which improved her blood pressure to 104/78 mmHg and oxygen saturation to 99%. Physical examination was significant for tachycardia, respiratory distress, and abdominal tenderness with appreciable Murphy's sign. A review of systems was notable for additional nausea, vomiting, palpitations, and leg swelling. Blood glucose level measured in the emergency room was 183, and her last hemoglobin A1c obtained a month prior by her primary care physician's office was 11.3. The initial troponin level was elevated at 85, with a repeat level decreased to 58. An electrocardiogram (ECG) revealed sinus tachycardia with no definitive evidence of ischemia, as depicted in Figure 1. D-dimer was elevated, but subsequent computed tomography (CT) of the chest with IV contrast was negative for pulmonary embolism and any acute pulmonary process. CT of the abdomen showed evidence of gallstones with concerns of cholecystitis, as depicted in Figure 2. Broad-spectrum IV antibiotics such as vancomycin and cefepime were consequently started due to possible sepsis thought to be secondary to acute cholecystitis. Blood and urine cultures were obtained, and general surgery was notified for consultation. Urine culture was negative; however, peripheral and chemotherapy port blood cultures revealed the presence of gram-negative bacillus, *Pantoea*.

Upon consultation with general surgery, the patient appeared to have symptomatic cholelithiasis and was recommended for immediate laparoscopic cholecystectomy. The procedure was completed without complication, and the patient tolerated the surgery well. During the postoperative period, sensitivity testing for *Pantoea *was completed and revealed susceptibility to cefepime, as depicted in Table 1. The patient was initially placed on cefepime, leading to improvement as the patient became afebrile with her white blood cell count trending down. After consultation with infectious disease, the patient was switched from cefepime to oral levofloxacin for 10 days. It was also recommended that the port be removed as it was thought to be the nidus of infection as well as causing local inflammation and itching. The patient continued to improve and was eventually discharged with home health. Upon follow-up, the patient is currently in remission for her breast cancer and is reportedly doing well.

**Table 1:** This table shows the results of the patient's sensitivity testing for Pantoea. The MIC refers to the lowest concentration of antibiotic necessary to inhibit bacterial growth. In this case, this particular isolate was only resistant to cefazolin, allowing for the patient to be treated with levofloxacin successfully. CSF: cerebrospinal fluid; MIC: minimum inhibitory concentration

Antibiotics	MIC	Susceptibility
Cefazolin	≤1 ug/mL	Resistant
Cefepime	≤1 ug/mL	Susceptible
Ceftriaxone (non-CSF)	≤0.25 ug/ml	Susceptible
Gentamicin	≤1 ug/mL	Susceptible
Imipenem	0.5 ug/mL	Susceptible
Levofloxacin	≤0.12 ug/mL	Susceptible
Piperacillin+tazobactam	8 ug/mL	Susceptible
Tobramycin	≤1 ug/mL	Susceptible
Trimethoprim+sulfamethoxazole	≤20 ug/mL	Susceptible

## Discussion

As previously mentioned, the genus *Pantoea* more commonly infects plants while having the potential to cause significant disease in humans. When *Pantoea* is isolated as a causative agent in human disease, this occurs most often in the setting of agricultural work-related exposure. Specifically, *Pantoea agglomerans*, the most common species present in the microbiota of cotton plants as well as grain dust, leads to a variety of respiratory disorders upon inhalation of the endotoxin and bacteria itself [4,5]. More rarely, *Pantoea* species present as an etiology of nosocomial infections, such as in our patient's case. 

In the nosocomial setting, *Pantoea* operates as an opportunistic pathogen by infecting immunocompromised people. Given that the most common route of infection in this setting is through medical devices, our patient having a chemotherapy port made her the ideal candidate. Based on previous case reports and literary reviews, there is no current documentation on person-to-person transmission of *Pantoea*. Transmission of *Pantoea* to humans has been reported to occur through interactions with the environment, whether it be through inhalation of organic dusts or contact with medical equipment in a hospital setting [2,3]. 

Additionally, *Pantoea* has been seen to develop resistance to multiple antibiotic classes such as fluoroquinolones, aminoglycosides, and cephalosporins. Research has suggested that *Pantoea* can form multiple routes of resistance including antibiotic efflux pumps and ATP-binding cassettes [6]. Fortunately, in our case, the species isolated from the patient's port only demonstrated resistance to cefazolin. This allowed for relatively accessible management of the patient with cefepime and, eventually, levofloxacin. While the isolate in this situation had several sensitivities, if *Pantoea* infections appear more frequently in a healthcare setting, there are concerns for increased multiple drug resistance, which could lead to challenges in future management. 

Shortly after the resolution of the patient's infection, she was found to be breast cancer-free. This finding could point to the potential antiproliferative effects of *Pantoea* species. A study isolated an exopolysaccharide produced by *Pantoea *known as levan, which has demonstrated potent antimicrobial and anti-cancer properties [7]. The polysaccharide is part of the greater LPS endotoxin that *Pantoea* secretes. The study revealed that the levan molecule produced cytotoxic effects on malondialdehyde (MDA) breast cancer and rhabdomyoma cell lines along with antioxidant properties and antiparasitic activity. While the mechanism of antiproliferation is still unknown, it is postulated that levan upregulates tumor suppressor genes like p53 and p27, halting the proliferation of cancer cells [7]. 

## Conclusions

Although rare, *Pantoea* can be a potential infectious agent in acute cholecystitis associated with bacteremia, especially in immunocompromised patients. Due to limited reports, this case will help guide the management of future *Pantoea* infections. The mainstay of treatment follows intuitive sepsis protocol: starting appropriate empiric antibiotic therapy to cover the most common nosocomial organisms, obtaining appropriate cultures to identify the causative organism, and then performing sensitivity testing to guide antibiotic therapy. Sensitivity testing is crucial for *Pantoea* infection since previously documented cases of *Pantoea*-related disease have demonstrated marked resistance to several antibiotic classes. 
